# Significance of the metastasis-inducing protein AGR2 for outcome in hormonally treated breast cancer patients

**DOI:** 10.1038/sj.bjc.6603065

**Published:** 2006-04-04

**Authors:** H E Innes, D Liu, R Barraclough, M P A Davies, P A O'neill, A Platt-Higgins, S de Silva Rudland, D R Sibson, P S Rudland

**Affiliations:** 1 Clatterbridge Cancer Research Trust, JK Douglas Laboratories, Clatterbridge Hospital, Wirral CH63 4JY, UK; 2 Cancer Tissue Bank Research Centre, University of Liverpool, Liverpool L69 7ZB, UK; 3 School of Biological Sciences, Biosciences Building, University of Liverpool, Crown Street, Liverpool L69 7ZB, UK

**Keywords:** AGR2 immunocytochemistry, patient survival, ER*α*-positive breast cancer

## Abstract

The anterior gradient protein-2 (AGR2) is inducible by oestrogen and itself can induce metastasis in a rat model for breast cancer. Here, a rabbit antibody to recombinant human AGR2 was used to assess its prognostic significance in a retrospective cohort of 351 breast cancer patients treated by adjuvant hormonal therapy. The antibody stains 66% of breast carcinomas to varying degrees. The percentage of positive carcinoma cells in tumours directly correlates with the level of AGR2 mRNA (Spearman's rank correlation, *P*=0.0007) and protein (linear regression analysis *r*^2^=0.95, *P*=0.0002). There is a significant association of staining of carcinomas for AGR2 with oestrogen receptor *α* (ER*α*) staining and with low histological grade (both Fisher's Exact test *P*<0.0001). In the ER*α*-positive cases, but not the ER*α*-negative cases, when subdivided into the separate staining classes for AGR2, there is a significantly progressive decrease in patient survival with increased staining (log rank test, *P*=0.006). The significant association of staining for AGR2 with patient death over a 10-year period (log rank test *P*=0.007, hazard ratio=3) only becomes significant at 6 years of follow-up. This may be due to the cessation of adjuvant hormonal therapy at an earlier time, resulting in adverse re-expression of the metastasis-inducing protein AGR2.

Molecules involved in metastasis offer a potential source for the identification of useful markers of prognosis for breast cancer, since the majority of deaths are attributable to the formation of secondary tumours at site or sites distant to the primary tumour (Kamby, 1990; EBCTCG, 2005). Recently, anterior gradient protein-2 (AGR2; previously hAG-2), human homologue of the *Xenopus laevis* cement gland protein, XAG-2, has been identified, by suppression subtraction hybridisation, as being expressed at a higher level in the human breast cancer cell line MCF-7 than in the benign cell line Human mammary 123 (Liu *et al*, 2005). Transfection of AGR2 cDNA into a benign rat mammary cell line, Rat mammary (Rama) 37, induced a metastatic phenotype *in vivo*, such that when the cells were injected into the mammary fat pads of syngeneic rats, the majority (77–82%) developed lung metastases (Liu *et al*, 2005). In similar experiments, the Rama 37 cells have been shown previously to be converted to a metastatic phenotype by genes encoding the proteins S100A4 (Davies *et al*, 1993) and osteopontin (Oates *et al*, 1996). Expression of both of these proteins, as measured by immunohistochemistry in the primary tumour, has been shown to correlate with survival in a cohort of patients with breast cancer, such that higher levels of expression act as a marker of poor prognosis (Rudland *et al*, 2000, 2002). However, human AGR2 has been reported previously to be expressed in the oestrogen receptor alpha (ER*α*)-positive cell line MCF-7 but not in an ER*α*-negative cell line (Thompson and Weigel, 1998), and preliminary studies in human breast cancers (Fletcher *et al*, 2003; Liu *et al*, 2005) also suggest that AGR2 is correlated with expression of ER*α*. There is, therefore, a potential inconsistency, in that ER*α* and related markers usually associated with a better outcome and markers of metastasis with a worse outcome for breast cancer patients. The aim of this study is to examine for the first time the expression of AGR2 in specimens of primary breast carcinomas so as to assess its relationship with other tumour variables and with patients' survival in a group of patients treated by hormonal therapy.

## MATERIALS AND METHODS

### Patients and specimens

Patients undergoing treatment for invasive breast cancer during the period 1982 to 1999 at the Royal Liverpool University Hospital were identified from the Department of Surgery (Martin *et al*, 1997; de Silva Rudland *et al*, 2006) and the Cancer Tissue Bank Research Centre, University of Liverpool (O'Neill *et al*, 2004). A total of 351 patients with stage I/II breast cancer were selected; staging investigations to exclude metastatic disease varied but generally included chest radiograph and liver function tests. They had been treated by surgery, with or without radiation, but had not received systemic chemotherapy. Median age was 64 years (range 31–89), their clinical, histological and molecular characteristics are summarised in Table 1. The vast majority received some form of adjuvant endocrine treatment. Clinical follow-up data were recorded by retrospective case-note review and from the Merseyside Cancer Registry. The outcome measure was overall survival, with data from surviving patients being censored at the date last seen. Median follow-up was 85.9 months (range 0.1–212). For statistical analysis, follow-up was limited to 10 years. Ethical approval for the study was obtained from all relevant bodies.

### Immunohistochemistry

Histological sections (4 *μ*m) were cut from the formalin-fixed, paraffin-embedded specimens and placed onto 3-aminopropyltriethoxysilane-coated slides and endogenous peroxidase activity was blocked using 3% (v v^−1^) hydrogen peroxide (H_2_O_2_) (Platt-Higgins *et al*, 2000). Oestrogen receptor *α* and progesterone receptor (PR) status was obtained from review of histopathology notes (O'Neill *et al*, 2004) where available, or was determined immunocytochemically (de Silva Rudland *et al*, 2006) as described previously (Platt-Higgins *et al*, 2000) using a cutoff of 5% to define the positive and negative groups. Immunohistochemical staining for AGR2 was performed with affinity-purified AGR2 antibody as previously described (Liu *et al*, 2005), with minor modifications. Slides were preincubated with 2% (w v^−1^) bovine serum albumin (BSA) in phosphate-buffered saline (PBS) for 1 h and then incubated with antibody to AGR2 (diluted 1 in 400 in 2% (w v^−1^) BSA in PBS) for 3 h. The bound antibodies were detected using biotinylated donkey anti-rabbit immunoglobulin (Amersham Biosciences, Bucks, UK) diluted 1 in 200 in 1% (w v^−1^) BSA in PBS and sections were incubated for 1 h. The bound antibodies were visualised as a brown stain by incubating the sections with 3,3′-diaminobenzidine (Sigma, Dorset, UK) and 0.075% (v v^−1^) H_2_O_2_. They were counterstained with Mayers' haemalum and mounted in DPX (Merck, Dorset, UK) (Rudland *et al*, 2002). The raising of the rabbit antibody against recombinant human AGR2 and its specificity have been described previously (Liu *et al*, 2005). Positive staining was abolished by prior incubation of the antiserum with 1 mg ml^−1^ human recombinant AGR2 (rAGR2) protein. Stained slides were analysed independently by two observers using light microscopy; the percentage of positively stained malignant cells was estimated by scanning the whole section at lower power and for at least 10 microscopic fields at × 200 magnification to ensure a representative sample. Staining for AGR2 was evaluated in six classes. These comprised: negative (<1% carcinoma cells stained), borderline (1–5% cells stained), intermediate (5–25% cells stained), moderate (25–50% cells stained), strong (50–75% cells stained) and very strong (75–100% cells stained). Where two-way analyses were performed, the borderline group was combined with the positive cases, leaving the negative cases as a separate category (unless otherwise stated).

In controls, the rabbit antiserum was preincubated with 700 *μ*g ml^−1^ human rAGR2 or rAGR3 prior to application to sections from positively staining specimens and a human AGR2 monoclonal antibody was incubated with sections from 20 different specimens chosen at random. The monoclonal antibody to AGR2 was raised against a peptide unique to the AGR2 sequence, which did not occur in AGR3 and failed to react with AGR3 protein (Liu D, Rudland PS, Barraclough R, unpublished results).

### Protein samples and Western blotting

The rAGR2 containing the histidine tag and a protease factor X cleavage site and the similarly engineered human recombinant AGR3 (rAGR3) proteins (Liu D, Rudland PS, Barraclough R, unpublished results) were produced and purified as previously described (Liu *et al*, 2005). The apparent molecular weights of 21 and 19 kDa, respectively, were consistent with 21 and 19.8 kDa calculated from the amino-acid sequences of the open reading frames of the cDNA inserts in the expression vectors used (Liu *et al*, 2005). Samples of human breast cancer specimens were frozen in liquid nitrogen and powdered in a pestle and mortar. They were homogenised in a Polytron homogeniser in a guanidinium isothiocyanate buffer and fractionated by centrifugation on a cushion of CsCl as described previously (Anandappa *et al*, 1994). The resultant supernatant protein fraction was dialysed against 10 mM NH_4_CO_3_, lyophilised, then redissolved in sample buffer containing 2%(w v^−1^) SDS, 2 mM phenylmethanesulphonyl fluoride, together with glycerol, *β*-mercaptoethanol and bromophenol blue. Soluble protein lysates from cell lines were obtained as described previously (Liu *et al*, 2005). Samples containing equal amounts of total proteins were resolved on 0.1% (w v^−1^) SDS, 12.5%(w v^−1^) polyacrylamide gels together with molecular weight markers, human rAGR2 and rAGR3 proteins. The proteins were electrotransferred onto Immobilon PVDF membranes (Millipore (UK) Ltd, Watford, UK) using a Bio-Rad semidry transfer apparatus (Bio-Rad Laboratories Ltd, Hertfordshire, UK). The membranes were incubated with buffer containing 5% (w v^−1^) nonfat dried milk for 1 h at room temperature, then with the affinity-purified, in-house rabbit polyclonal anti-human AGR2 antibody as described previously (Liu *et al*, 2005). In some experiments, 1 mg ml^−1^ human rAGR2 was present to provide a blocked antibody control. After washing and incubating with anti-rabbit horseradish peroxidase-conjugated IgG, the membranes were washed and exposed to the Supersignal West Pico Chemiluminescent Substrate (Pierce Biotechnology Inc., Perbio Sciences, Cramlington, Northumberland, UK) according to the manufacturer's instructions. The chemiluminescent signals were collected and analysed using the ChemiDoc XRS system (Bio-Rad). The membranes were reprobed with rabbit polyclonal *β*-actin antibody (New England BioLabs (UK) Ltd, Hertfordshire, UK) to ensure equal protein loading as described previously (Liu *et al*, 2005). Statistical analyses of Western blotting and immunocytochemical staining were carried out by least-squares regression using Arcus Pro-Stat Dos version 3.28 software (Medical Computing, Aughton, UK).

### Reverse transcription (RT)–PCR analysis

RNA of suitable quality was available for 84 cases and MCF-7 cell line RNA was used as a control. RT was performed as described previously (Livak and Schmittgen, 2001; O'Neill *et al*, 2004). Quantitative PCR was performed on a Bio-Rad Icycler PCR Real-Time PCR machine using 2 *μ*1 of a 1 : 20 dilution of cDNA per reaction (equivalent to cDNA from approximately 2.5 ng of total RNA). Reactions included 1 × IQ SYBR Green Supermix (Bio-Rad) and 1 *μ*M of each PCR primer: AGR2-Forward GAG CCG ATA TCA CTG GAA GA and AGR2-Reverse CAA GGC CTG ACA GAC AGA AG; or HPRT-Forward GTG TTG GAT ATA AGC CAG ACT TTG and HPRT-Reverse AAC TCA ACT TGA ACT CTC ATC TTA GGC. The PCR reaction consisted of a hot-start *Taq* Polymerase activation step of 95°C for 3 min, followed by either 50 cycles at 94°C for 30 s/62°C for 90 s for AGR2, or 36 cycles of 94°C for 30 s/64°C for 60 s for HPRT. Relative expression of mRNA for each gene was calculated relative to MCF-7 cell line RNA, using the *δδC*t method (Livak and Schmittgen, 2001) correcting for the control gene (HPRT). The identity of PCR products as AGR2 and not AGR3 was confirmed by DNA sequence analysis as described previously ((Livak and Schmittgen, 2001; O'Neill *et al*, 2004).

### Statistical analysis

Statistical analyses were performed using the SPSS® package (Windows, v.11). The association of immunohistochemical staining for AGR2 with other tumour variables was assessed using Fisher's exact test, two-sided values of *P* are given. The degree of agreement between observers was assessed using the kappa (*κ*)-statistic; a value of >0.61 was taken to be a satisfactory agreement (Altman, 1991). Curves for overall survival were generated using the Kaplan–Meier method for censored data, with surviving patients' data being censored at the date of their last clinic visit. Curves from different groups of patients were compared using both the log rank test and the Wilcoxon (Gehan) statistic. Unadjusted hazard ratios (HRs)±95% confidence intervals (CIs) were obtained using Cox's univariate analysis, as described previously (Rudland *et al*, 2002). Spearman's rank correlation was used as a measure of association between abundance of mRNA and the degree of immunohistochemical staining, and the Student's *t*-test and the Mann–Whitney *U*-test were used to compare the level of AGR2 mRNA between cases which were AGR2 positive and those that were AGR2 negative by immunohistochemistry. Cox's regression model was used for multivariate survival analysis (Altman, 1991).

## RESULTS

### Immunohistochemical staining for AGR2

**Figure 1:**
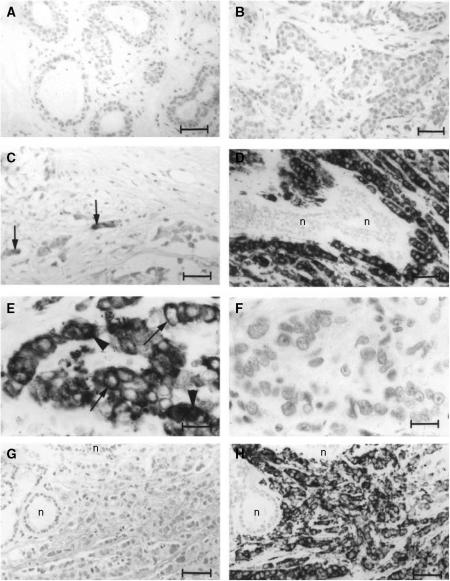
Immunocytochemical staining for AGR2. Antiserum to AGR2 was incubated with histological sections of specimens from (**A**) normal breast or from (**B**–**D**) primary tumours of different breast carcinomas showing: (**B**) unstained (−); (**C**) borderline (±); (**D**) strong immunocytochemical staining of the carcinoma cells (+++). Arrows point to the occasional stained cell in **C**; n is unstained normal breast tissue in **D**. (**E**) is a higher magnification of **D** showing strong immunocytochemical staining of the cytoplasm (arrowheads) and membranous region (arrows) of the carcinoma cells. (**F**) Antiserum to AGR2 preincubated with pure human rAGR2 applied to an adjacent serial section to that in **E** showing no immunocytochemical staining. (**G**) Antiserum to AGR2 preincubated with pure human rAGR2 showing no immunocytochemical staining over a larger field at lower magnification to that in **F**. (**H**) Antiserum to AGR2 preincubated with pure human rAGR3 showing strong immunocytochemical staining of the carcinoma cells in a section adjacent to that in **G**, the normal breast tissue n is unstained. Magnification **A**–**D**, **G**, **H** × 230; **E**, **F** × 580. Bars **A**–**D**, **G**, **H** 50 *μ*m; **E**, **F** 20 *μ*m.

Immunocytochemical staining of normal breast tissue was usually negligible (Figure 1A). Staining of primary breast carcinomas for AGR2 showed great variation from tumour to tumour in the proportion of cancer cells staining, ranging from none to >90% (Figure 1B–D). The staining was mainly cytoplasmic and membranous (Figure 1E). There was some intraobserver variability, but when tumours were divided into the broad categories of positively and negatively stained tumours there was agreement in 97.2%, corresponding to a *κ*-score of 0.94. There was a similar small variation in the assessment of the same histological section by two observers with agreement in 97.4% of cases, corresponding to a *κ*-score of 0.94 when divided into the broad categories of negative and positive. For the specimens where there was disparity, a consensus score was agreed. Overall, of the 351 cases, 120 (34.2%) were classified as unstained, defined as <1% of carcinoma cells stained, 61 (17.4%) were ‘borderline’ stained (1–5% carcinoma cells stained) and the remaining 170 (48.4%) were stained to some degree by the polyclonal antibody to the AGR2 protein. These were further subdivided into classes of 103 (29.3%) intermediate (i.e. 5–25% cells stained), 40 (11.4%) moderate (i.e. 25–50% cells stained), 20 (5.7%) strong (i.e. 50–75% stained) and 7 (2.0%) very strong (i.e. 75–100% cells stained). For 20 specimens chosen at random, the same staining classification was applied to staining obtained with a MAb specific for AGR2 which did not react with AGR3 (not shown). Prior incubation of the rabbit antiserum to AGR2 with pure human rAGR2 (Figure 1F and G), but not pure human rAGR3 (Figure 1H), abolished staining completely.

### Western blotting and RT–PCR for AGR2

**Figure 2:**
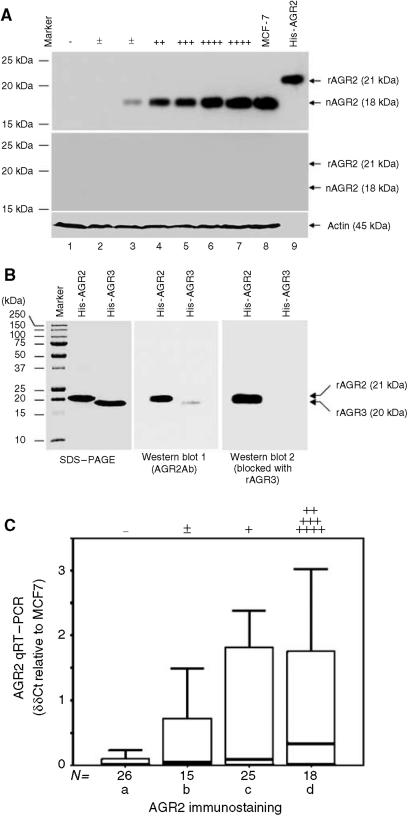
Detection of AGR2 by Western blotting and RT–PCR. (**A**) Protein samples (20 *μ*g) from invasive carcinomas of the following classes of immunocytochemical staining for AGR2: unstained (−; lane 1), borderline (±; lanes 2 and 3), moderate (++; lane 4), strong (+++; lane 5), very strong (++++; lanes 6 and 7), and 10 *μ*g of protein from human breast cancer cell line, MCF-7 (lane 8) or 0.5 *μ*g of purified His-tagged rAGR2 (lane 9) were subjected to polyacrylamide gel electrophoresis and blotted onto Immobilon PVDF membranes as described in Materials and Methods. The membranes were incubated overnight at 4°C with one of the following: a 1 : 400 dilution of rabbit polyclonal anti-human AGR2 (**A**, upper panel), the same amount of rabbit polyclonal anti-human AGR2 preincubated overnight at 4°C with 1 mg ml^−1^ human rAGR2 (**A**, middle panel) and 1 : 1000 dilution of rabbit anti-*β*-actin antibodies (**A**, lower panel). The positions of human natural AGR2 (nAGR2), recombinant His-tagged AGR2 (rAGR2) and actin are shown on the right-hand side of the panels and those of molecular weight markers are shown on the left-hand side of the panels. (**B**) 5 *μ*g (**B**, SDS-PAGE panel) or 0.5 *μ*g (**B**, Western blot panels) of purified His-tagged rAGR2 and His-tagged rAGR3 were subjected to 12.5% (w v^−1^) polyacrylamide SDS gel electrophoresis and stained with Coomassie blue G250 (**B**, SDS-PAGE panel) or blotted onto an Immobilon PVDF membrane (**B**, Western blot panels) as described in Materials and Methods. The membrane was incubated with one of the following: a 1 : 500 dilution of rabbit polyclonal anti-human AGR2 (**B**, Western blot 1 (AGR2 Ab) panel), the same amount of rabbit polyclonal anti-human AGR2 preincubated overnight at 4°C with 1 mg ml^−1^ human rAGR3 protein (**B**, Western blot 2 (blocked with rAGR3) panel), and the bound antibodies visualized as above. The positions of rAGR2 and rAGR3 are shown on the right-hand side of the panel and those of molecular weight markers are shown on the left-hand side. (**C**) Relative expression of AGR2 mRNA is shown in four categories of breast tumours: *a*, patients with carcinomas classified as unstained (−); *b*, borderline (±); *c*, intermediate (+) or *d*, moderate to very strong staining for AGR2 (++, +++ or ++++). AGR2 mRNA was determined by qRT–PCR and is shown relative to that in MCF-7 cells (*δδ*Ct relative to MCF-7) as described in Materials and Methods. The box represents the interquartile range, the line across the box indicates the median and the whiskers extend from the box to the highest and lowest values (excluding outliers and extreme points).

Antiserum to human AGR2 detected a band of 18 kDa in Western blots of extracts from primary breast carcinomas that were positive in immunocytochemical staining for AGR2 (Figure 2A). This band corresponded in size to that of AGR2 in MCF-7 cells and its appearance was abolished by prior incubation of the antiserum with human rAGR2 (Figure 2A). The histidine-tagged human rAGR2 protein containing a protease factor X cleavage site ran at an apparent molecular weight of 21 kDa (Figure 2A). All extracts contained approximately equal amounts of the constitutively expressed protein. In seven samples chosen at random, there was a significant correlation between the level of immunodetectable AGR2 by Western blotting and the percentage of cells immunocytochemically stained for AGR2 (linear regression analysis *r*^2^=0.95, *P*=0.0002). The antiserum to human AGR2 reacted strongly with His-tagged human rAGR2, but only weakly with the 19 kDa His-tagged human rAGR3, the ratio of intensities AGR2 : AGR3 being 10.2±1.3 (mean±s.d.) for equal amount of recombinant protein loaded onto the gel (Figure 2B). A band corresponding to normal AGR3 was not detected in human cell extracts with this antibody (Figure 2A).

Quantitative RT–PCR was performed on 84 of the above cases. The relative abundance of AGR2 mRNA correlated significantly with the class score for immunocytochemically detectable AGR2 protein in the cancer cells (Spearman's rank correlation statistic 0.36; *P*=0.0007). Furthermore, significantly higher AGR2 mRNA expression was seen in those cases that were AGR2 positive by immunohistochemistry compared to those that were AGR2 negative (Student's *t*-test, *P*=0.010; Mann–Whitney *U*-test, *P*=0.001; Figure 2C). Thus, the percentage of carcinoma cells stained immunocytochemically in the primary tumours was a reasonable reflection of the level of expression of AGR2 mRNA and protein.

### Association of staining for AGR2 with other tumour variables

The presence of immunocytochemical staining for AGR2 was cross-tabulated with the established prognostic factors of tumour size, nodal status, histological grade, lymphovascular invasion, ER*α* and PgR status (Table 2). For these analyses, the borderline staining group was combined with the positive staining group for AGR2, that is, using a 1% cutoff of the carcinoma cells staining (Materials and Methods). Positive staining for AGR2 was significantly correlated with that for the ER*α* receptor (Fisher's Exact test, *P*<0.0001) and with that for the PgR receptor (Fisher's Exact test, *P*=0.0002). Staining for AGR2 was also significantly associated with low tumour grade (Fisher's Exact test, *P*<0.0001). As expected, ER*α* positivity was significantly associated with low tumour grade (Fisher's Exact test, *P*<0.0001): 177 of 220 (80.5%) ER*α*-positive tumours were grade 1 or 2, compared to 42 of 115 (36.5%) ER*α*-negative tumours. Positive staining for AGR2 was not significantly associated with tumour size, nodal status or the presence of lymphovascular invasion (Table 2). There was also no significant association between positive staining for ER*α* and either tumour size or nodal status (not shown). If the borderline staining group were combined with the negative staining group (i.e. using a 5% cutoff) for AGR2, the same significant associations were seen. Within the ER*α*-negative group, there was a positive correlation between the degree of staining for AGR2 (>1% carcinoma cells stained) and the degree of staining for PgR (*n*=47 cases; Spearman's correlation coefficient 0.48; *P*=0.0007).

### Association of AGR2 with patient survival

**Figure 3:**
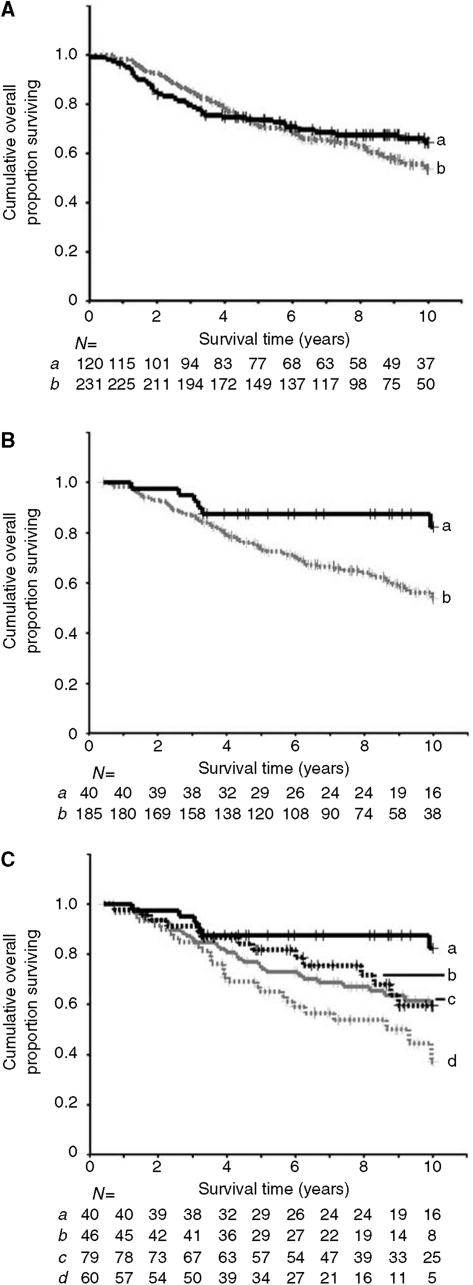
Association of immunocytochemical staining for AGR2 with overall survival of patients using a 1% cutoff between the two staining classes for (**A**) all cases, (**B**) ER*α*-positive cases or (**C**) by degree of immunostaining for AGR2 in ER*α*-positive cases. In (**A** and **B**), the cumulative proportion of surviving patients as a fraction of the total for each year after presentation for either *a* patients with carcinomas classified as negatively staining (black, unbroken line) or *b* positively staining (grey broken line) for AGR2 is shown. In (**A**), there were 81 censored observations in *a* and 139 in *b*. The cumulative proportions surviving were *a*, 0.71 at 5 years (standard error (s.e.)=0.04) and 0.64 at 10 years (s.e.=0.05) and *b*, 0.69 at 5 years (s.e.=0.06) and 0.54 at 10 years (s.e.=0.04). In (**B**), there were 34 censored observation in *a* and 114 in *b*. The cumulative proportions surviving were *a* 0.87 at 5 years (s.e.=0.05) and 0.82 at 10 years (s.e.=0.07) and *b* 0.81 at 5 years (s.e.=0.06) and 0.60 at 10 years (s.e.=0.09). In (**C**), the cumulative proportion of surviving patients with ER*α* positive primary tumours for *a* patients with carcinoma cells classified as unstained (black, unbroken line), *b* borderline (±, black broken line), *c* intermediate (+, grey unbroken line) or *d* moderate to very strong staining for AGR2 (++, +++ or ++++, grey broken line) is shown. There were 34 censored observations in *a*; 32 in *b*; 50 in *c* and 32 in *d*. The cumulative proportions surviving were *a* 0.87 (s.e.=0.05); *b* 0.81 (s.e.=0.06), *c* 0.73 (s.e.=0.05) and *d* 0.59 (s.e.=0.07) at 5 years; and *a* 0.82 (s.e.=0.07); *b* 0.60 (s.e.=0.09); *c* 0.59 (s.e.=0.06) and *d* 0.39 (s.e.=0.09) at 10 years. In all three panels, censored observations are denoted by vertical lines and the number of surviving patients in each subgroup (*N*) at 12 monthly intervals is shown below each panel.

In the whole group, there was no significant association between staining for AGR2 and patient survival (Figure 3A log rank test, *P*=0.33; Wilcoxon test, *χ*^2^=0.05, 1 d.f., *P*=0.82). However, when ER*α*-positive cases were considered separately (*n*=225), there was a strong negative correlation between the presence of AGR2 staining (using a 1% cutoff of the carcinoma cells stained) and patient survival, with patients whose tumours were AGR2 positive having significantly poorer survival than those whose tumours were AGR2 negative (Figure 3B; log rank test, *P*=0.007; Wilcoxon test, *χ*^2^=6.0, 1d.f., *P*=0.01). Median survival for this ER*α* positive cohort has not yet been reached for either AGR2-positive or AGR2-negative patients. The HR for survival of patients with AGR2-positive tumours compared to AGR2-negative tumours was 3.0 (95% CI 1.3–6.9). This difference in survival is a relatively late effect, with the survival curves beginning to diverge at approximately 40 months of follow-up, and the difference in overall survival becoming significant at 6 years. If the borderline staining group were combined with the negative staining group (i.e. using a 5% cutoff of the carcinoma cells stained), a significant association between staining for AGR2 and patient demise was also observed (log rank test, *P*=0.01; Wilcoxon test, *χ*^2^=6.2, 1 d.f., *P*=0.01) with an unadjusted HR of 1.9 (95% CI 1.1–3.2). When the ER*α*-positive cases were further divided into separate classes based on the percentage of carcinoma cells stained (<1%, 1–5, 5–25 and >25% positive cells), the four curves showed significantly progressively poorer survival (Figure 3C; log rank test, *P*=0.006; Wilcoxon test *χ*^2^=10.12, 3 d.f., *P*=0.02). Notably, cases with <1% cells staining had significantly better survival than cases with 5–25% positive cells (log rank test, *P*=0.03; Wilcoxon test *χ*^2^=4.2, 1 d.f., *P*=0.04) and cases with >25% positive cells staining (log rank test, *P*=0.0006; Wilcoxon test *χ*^2^=8.7, 1 d.f., *P*=0.003). When ER*α*-negative cases were considered separately, there was no significant association between staining for AGR2 and survival (log rank test, *P*=0.85; Wilcoxon test *χ*^2^=0.2, 1 d.f., *P*=0.66).

### Association of other tumour variables and AGR2 with patient survival

The established prognostic markers behaved in the expected manner with respect to patient overall survival, with positive nodal status, larger tumour size and high histological grade all associated with significantly poorer patient survival at 10 years of follow-up (log rank test, all *P*<0.0001). Oestrogen receptor *α*-negative status was not significantly associated with poorer patient survival at 10 years of follow-up (log rank test, *P*=0.1), as the survival curves had begun to converge, but was statistically significant at earlier time points (from 18 months to 8 years, log rank rest, *P*<0.04, e.g. at 3 years *P*=0.0006).

Within the ER*α*-positive group of patients, the association between immunocytochemical staining for AGR2 (at the 1% cut-off level) and patient survival was assessed further within subgroups defined by the other tumour variables. Staining for AGR2 was associated with poorer survival of patients with smaller tumours (T1 tumours *n*=117; log rank test, *P*=0.007; Wilcoxon test *χ*^2^=7.6, 1 d.f., *P*=0.006), but not with patients with larger tumours (*T*⩾2, *n*=94, log rank test, *P*=0.6; Wilcoxon test *χ*^2^=0.02, 1 d.f., *P*=0.9). As expected, the majority of the ER*α*-positive tumours were grades 1 or 2 (*n*=177) and within this subgroup staining for AGR2 was also associated with poorer patient survival (log rank test, *P*=0.009; Wilcoxon test *χ*^2^=5.8, 1 d.f.; *P*=0.02). In the small number of patients with ER*α*-positive grade 3 tumours (*n*=43), there was a trend for AGR2 to be associated with poorer survival, but this did not reach statistical significance (log rank test, *P*=0.1; Wilcoxon test *χ*^2^=1.9, 1 d.f., *P*=0.2). There was no significant difference in patient survival by AGR2 staining in ER*α*-positive patients when subdivided by nodal status (node negative, *n*=94; log rank test, *P*=0.34; Wilcoxon test *χ*^2^=0.56, 1 d.f., *P*=0.5; node positive, *n*=63; log rank test, *P*=0.46; Wilcoxon test *χ*^2^=0.03, 1 d.f., *P*=0.9). In Cox's multivariate analysis of the 114 patients available with full data sets (size, grade, nodal status, presence or absence of lymphovascular invasion and AGR2 staining) in the ER*α*-positive group, only nodal status was independently significantly correlated with patient survival (*χ*^2^=6.75, 1 d.f., *P*=0.009). The AGR2 was significantly associated with patient survival when considered pair-wise with tumour size (AGR2 HR 2.7, 95% CI 1.2–6.2, *P*=0.02, size HR 1.6, 95% CI 1.02–2.6, *P*=0.04) or pair-wise with grade (AGR2 HR 3.6, 95% CI 1.5–8.4, *P*=0.003, grade HR 2.5 95% CI 1.5–4.2, *P*=0.001).

## DISCUSSION

The developmentally related protein AGR2 has been shown, in a rat mammary model, to induce a metastatic phenotype (Liu *et al*, 2005). Preliminary studies have also indicated that expression of this protein is correlated with ER*α* expression in human breast cancer cell lines (Thompson and Weigel, 1998; Liu *et al*, 2005) and clinical specimens (Fletcher *et al*, 2003). In order to assess the relationship of AGR2 with other tumour variables including ER*α* and with patients' survival, we have examined the immunohistochemical expression of AGR2 in specimens of 351 primary breast carcinomas taken from patients who received no chemotherapy, but the vast majority (93%) of whom were given some form of endocrine treatment, usually tamoxifen post operatively (Table 1). Immunocytochemical staining was almost completely restricted to the cytoplasm and membranous region of malignant cells, and there was virtually no staining for AGR2 of normal host parenchymal or stromal tissues, consistent with previous studies (Fletcher *et al*, 2003; Liu *et al*, 2005). Overall, 64.8% of breast cancers stained for AGR2 using a 1% cutoff to differentiate the negatively and positively staining tumours. This value is a little lower than the staining levels found in pilot studies: 75% using the same antibody (Liu *et al*, 2005) and 83% using a different antibody (Fletcher *et al*, 2003).

The specificity of staining for AGR2 in the clinical samples has been verified in 20 randomly selected cases by obtaining the same results when the concentration of antibody is increased five-fold, when another batch of antiserum raised in a different rabbit is employed and by the abolition of staining by prior incubation of the antiserum with pure recombinant protein. Moreover, when tested by Western blotting techniques, the rabbit antiserum reacts with only a single band of 18 kDa in extracts of selected positively staining carcinomas and in the breast cancer cell line MCF-7, in agreement with previous results for AGR2 (Liu *et al*, 2005). Although the rabbit antibody to AGR2 also crossreacts to a very limited extent by Western blotting with AGR3, the immunocytochemical staining observed arises almost exclusively from AGR2 and not from any potential AGR3 for the following reasons: (1) The MAb which is entirely specific for AGR2 and does not crossreact with AGR3 yields the same staining classification for 20 specimens selected at random. (2) Prior incubation of the rabbit antiserum with pure human rAGR3 fails to abolish any staining. (3) No band corresponding to AGR3 is detectable in human cell extracts with this antibody.

Using the rabbit antibody to AGR2, the interobserver and intraobserver variability in immunocytochemical staining observed between the negatively and positively staining groups is sufficiently small (2.6 and 2.8%, respectively) not to affect appreciably the reported results. The different classes of immunocytochemical staining of the carcinoma specimens based on the proportion of AGR2 immunoreactive carcinoma cells may also reflect the levels of expressed AGR2 protein, because the levels of AGR2 immunoreactive protein, as determined by Western blotting, are linearly correlated with the percentage of stained carcinoma cells by immunocytochemistry in the limited number of specimens studied. Moreover, expression of AGR2 as measured by immunocytochemistry correlates well with quantitative RT–PCR measurement of the abundance of AGR2 mRNA. This indicates that mRNA production is probably the primary control point for expression of AGR2 and is consistent with the mRNA-based subtractive hybridisation approach used to first identify AGR2 as a potential marker of aggressive ER*α*-positive tumours (Liu *et al*, 2005).

In specimens from the 351 breast carcinomas, there was a strong positive association between the presence of immunocytochemical staining for AGR2 and for ER*α*. Moreover, within the ER*α*-negative group of tumours, staining for AGR2 was positively correlated with staining for PgR. These findings are consistent with our cell line data in which AGR2 mRNA is present at much higher levels (seven-fold greater), in oestrogen-responsive MCF-7 cells grown in the presence of oestrogen than in oestrogen-depleted conditions (Liu *et al*, 2005) and with other studies (Thompson and Weigel, 1998; Fletcher *et al*, 2003), and are strongly indicative of AGR2 being an ER-dependent gene. In turn, the relationships between AGR2 and other tumour variables reflected this strong association with ER*α* positivity; thus, staining for AGR2 is associated with tumours of lower grade but not with tumour size, nodal status or the presence of lymphovascular invasion. However, since almost a third of ER*α*-negative cases stained for AGR2 and not all ER*α*-positive cases stained for AGR2, other factors must also influence expression of AGR2. Previously we had shown that AGR2 can increase adhesion of Rama 37 cells either when directly added or when overexpressed after transfection with an expression vector for AGR2 (Liu *et al*, 2005). However, no attempts have been made to investigate the effect of oestrogen on cellular adhesion in MCF-7 cells, since the production of another adhesion-inducing molecule, osteopontin (Moye *et al*, 2004), in addition to AGR2, is also stimulated by oestrogen (El-Tanani *et al*, 2001), so complicating interpretation of the results.

The finding that in the whole patient group there is no significant association between staining for AGR2 and patient outcome is also consistent with a role for AGR2 not solely related to ER*α*, since ER*α*-positive status is itself associated with a better patient outcome. That AGR2 has potential as a marker independent of ER*α* is confirmed by our findings when the ER*α*-positive and ER*α*-negative patient subgroups are considered separately. In the ER*α*-positive group, positive staining for AGR2 is associated with significantly poorer patient survival than that of the AGR2-negative group, with an unadjusted HR of 3. Moreover, when the ER*α*-positive cases are subdivided into separate classes by increasing proportion of carcinoma cells staining for AGR2, the survival curves showed progressively poorer survival. Thus, there appears to be a ‘dose response’ relationship between the % cell staining for AGR2 and adverse impact on patient outcome in the ER*α*-positive group of tumours. The fact that overexpression of AGR2 in benign rat mammary cells causes them to metastasise in syngeneic rats *in vivo* (Liu *et al*, 2005), but the human breast cancer cell line isolated from a metastatic plural effusion (Soule *et al*, 1973) fails to exhibit aggressive behaviour *in vitro* and to metastasise *in vivo* (Clarke *et al*, 1990) despite expressing AGR2, is at first sight surprising. However, MCF-7 cells when exposed to high levels of oestrogens which induce increased levels of AGR2 (Liu *et al*, 2005) assume a more aggressive phenotype *in vitro* and can form tumours and metastases in *nu nu* mice *in vivo* (Clarke *et al*, 1994; Kern *et al*, 1994). Moreover, the immunodeficient rodent is a poor model for breast cancer metastasis, since T-cell-depleted rats fail to allow the formation of tumours and metastases from a rat cell line that is highly metastatic in the same immune-competent intact rats (Rudland *et al*, 1989).

The differential impact of staining for AGR2 on prognosis in ER*α*-positive and ER*α*-negative patients is in contrast to the adverse effect of staining for S100A4 and OPN on the survival of patients with breast cancer which are apparent in both ER*α*-positive and ER*α*-negative subgroups (Rudland *et al*, 2000, 2002). These proteins were identified as inducers of the metastatic phenotype in experiments similar to those performed for AGR2 (Davies *et al*, 1993; Oates *et al*, 1996). However, upon transfection of an expression vector for AGR2 into parental Rama 37 cells and their transplantation into syngeneic rats, the primary tumours appear on average with an increased latency compared to that for S100A4-transfected, osteopontin-transfected or the parental Rama 37 cells (Davies *et al*, 1993; Oates *et al*, 1996; Liu *et al*, 2005). Nevertheless, this average slower tumour growth does not hinder the ability of AGR2-transfected cells to form metastases, it merely delays it. It is tempting to speculate that a similar effect of AGR2 in human tumours may account for the late effect on patient outcome found in the ER*α*-positive group in this study; the survival curves do not begin to separate until approximately 40 months and become significant only at 6 years. Since breast cancer relapses in ER*α*-positive patients often occur late (Saphner *et al*, 1996), this finding may be of clinical relevance, particularly in relation to consideration of extended adjuvant endocrine therapy in such patients. It may further be suggested that the absence of an impact of staining for AGR2 on prognosis in ER*α*-negative patients may be related to the higher proliferation rate of their tumours and tendency to earlier metastasis, with other genes/gene products having a greater influence on patient outcome.

An additional explanation for the differential prognostic impact of AGR2 on ER*α*-positive and ER*α*-negative subgroups of tumours may relate to the treatment received by these patients. The majority have received adjuvant endocrine therapy (predominantly tamoxifen), which would be expected to have an effect on ER*α*-positive but not ER*α*-negative patients. AGR2 mRNA is present at much higher levels (seven-fold greater) in oestrogen-responsive MCF-7 cells grown in the presence of oestrogen than in cells grown in oestrogen-depleted conditions (Liu *et al*, 2005). Therefore, it may be expected that the expression of AGR2 would be suppressed by the anti-oestrogenic effects of tamoxifen. In this group of patients, tamoxifen was usually given for between 2 and 5 years and therefore the apparent separation of the survival curves at around 3–4 years in ER*α*-positive patients would be consistent with the removal of adjuvant hormonal therapy resulting in the adverse re-expression of AGR2.

Our results demonstrate that expression of AGR2, as measured by immunohistochemistry, is associated with poor outcome in patients with ER*α*-positive breast cancers. Further studies are required to characterise this relationship, particularly with regard to how ER*α* induces the expression of AGR2 and the exact mechanism of AGR2 in the metastatic process. It is to be hoped that knowledge of expression of AGR2 may, in the future, help to inform treatment decisions for patients with ER*α*-positive breast cancers in the adjuvant or extended adjuvant settings.

## Figures and Tables

**Table 1:** Clinical, histological and molecular characteristics of primary breast carcinomas

**Characteristic^a^**	**Group**	**No^b^**	**%^c^**
Histology	Invasive ductal	293	83.5
	Invasive lobular	28	8.0
	Other	30	8.5
			
Extent of surgery	Wide local excision	249	70.9
	Mastectomy	75	21.4
	Unknown	27	7.7
			
Endocrine therapy	No	14	4.0
	Yes	327	93.2
	Unknown	10	2.8
			
Radiotherapy	No	194	55.3
	Yes	139	39.6
	Unknown	18	5.1
			
Histological grade	I	80	22.8
	II	144	41.0
	III	119	33.9
	Unknown	8	2.3
			
Tumour size	Up to 2 cm (T1)	181	51.6
	>2 cm to 5 cm (T2)	142	40.5
	>5 cm (T3)	9	2.6
	Unknown	19	5.4
			
Nodal status	Negative	141	40.2
	Positive	105	29.9
	Unknown	105	29.9
			
Lymphovascular invasion	Negative	122	34.8
	Positive	107	30.5
	Unknown	122	34.8
			
ER*α* status	Negative	117	33.3
	Positive	225	64.1
	Unknown	9	2.6
			
PgR status	Negative	79	22.5
	Positive	60	17.1
	Unknown	212	60.4

a Defined in ‘Materials and Methods’; ER*α*=oestrogen receptor *α*; PgR=progesterone receptor.

b Number of patients.

c Percentage of total patients, out of a total of 351.

**Table 2:** Association of immunohistochemical staining for AGR2 with other tumour variables

**Tumour variable^a^**	**AGR2-positive^b^ no. (%)**	**AGR2-negative^b^ no. (%)**	**Statistical significance^c^**
Grades 1, 2	178 (79.1)	46 (39.0)	
Grade 3	47 (20.9)	72 (61.0)	<0.0001
			
T1	120 (55.3)	61 (53.0)	
T2, T3	97 (44.7)	54 (47.0)	0.73
			
ER*α* negative	38 (17.0)	79 (66.4)	
ER*α* positive	185 (83.0)	40 (33.6)	<0.0001
			
Node negative	96 (57.5)	45 (57.0)	
Node positive	71 (42.5)	34 (43.0)	1
			
LVI negative	84 (54.2)	38 (51.4)	
LVI positive	71 (45.8)	36 (48.6)	0.78
			
PgR negative	44 (46.3)	35 (79.5)	
PgR positive	51 (53.7)	9 (20.5)	0.0002

a Grades, 1 and 2 *vs* 3; tumour size, T1 *vs* T2 and T3; oestrogen receptor *α* negative (ER*α*-negative) *vs* positive; lymph nodes containing no tumour, node negative *vs* positive; no lymphovascular invasion (LVI) negative *vs* positive; progesterone receptor-negative (PgR-negative) *vs* positive.

b Numbers (percentage) of patients with tumours staining (positive) or not staining (negative) for AGR2.

c Probability, *P*, from Fisher's Exact test.
